# Validating the underpinnings of water corticosterone measurement for aquatic amphibians

**DOI:** 10.12688/f1000research.157055.1

**Published:** 2025-01-09

**Authors:** Tessa E Smith, Andrew M Holmes, Christopher J Emmans, Robert Coleman, Charlotte A Hosie

**Affiliations:** 1 Amphibian Behaviour and Endocrinology Group, School of Natural Sciences, University of Chester, Department of Biological Sciences, Chester, England, CH1 4BJ, UK

**Keywords:** water-borne, corticosterone, Xenopus, welfare, husbandry, amphibian

## Abstract

**Background:**

Good animal welfare is important ethically but also to ensure animals provide valid scientific models. Despite thousands of amphibians in research laboratories there is minimal quantitative evidence pertaining to their management and welfare. This study validated methods to non-invasively measure corticosterone, the amphibian ‘stress’ hormone, from tank water to provide a robust and reliable welfare assessment tool.

**Methods:**

We report experiments (A) that evaluate parameters linked to the performance of our biochemical extraction methods for waterborne corticosterone and, importantly, associated sampling procedures. We evaluate appropriate sampling water type, sampling vessel, filtration methods, potential degradation of waterborne corticosterone over time and the impact of sampling procedures on animal corticosterone levels. We wanted to determine sampling parameters that yielded the least background corticosterone and had minimum negative impact on the animals. The second series of experiments (B) evaluated parameters linked to the biology of *Xenopus*, including the influence of circadian rhythm, sex and snout-vent length on waterborne corticosterone levels, since fundamental knowledge of a species’ biology is essential for designing robust experiments and in the interpretation of the results.

**Results:**

We propose collecting corticosterone samples in deionised water in either plastic or glass containers. The filtering process does not impact the amount of corticosterone measured in the water sample. Levels of corticosterone collected in the water change over a 48-hr period so we advocate standardising time from hormone collection to storage at - 20 °C. Repeated transfer of frogs to sampling containers does not increase corticosterone, suggesting our methods are not cumulatively stressful. Corticosterone levels were not impacted by circadian phase, sex or snout-vent length.

**Conclusion:**

We have developed and validated robust methods to quantify waterborne corticosterone. We hope they provide a template for researchers wishing to develop methods to measure waterborne corticosterone in aquatic amphibians.

Research highlights **Scientific benefits**

Development and validation of robust, reliable non-invasive methods to measure waterborne corticosterone in aquatic amphibians that do not cumulatively increase Hypothalamic-pituitary-inter-renal (HPI) function.

**3Rs benefits**

Our welfare quantification methods are non-invasive and do not cumulatively raise HPI activity or reduce body mass (a downstream indicator of stress).

The methods can be used to optimise captive animal welfare i.e., minimise corticosterone release by refining the housing and management protocols for captive aquatic amphibians.

Animals with good welfare make better scientific subjects compared to animals with poor welfare since they produce more reproducible, reliable scientific data, hence enabling reduction of experimental sample sizes and animal use.

**Practical benefits**

Methods described here allow a timely non-invasive measurement of stress hormones by defining a robust sampling method and an appropriate time course.

**Current applications**

The measurement of stress hormones in three species of captive/laboratory amphibians in order to refine housing and husbandry protocols to maximise good welfare.

The methods have also been validated for three species of UK aquatic amphibians in the field to assess the impact on the hypothalamic-pituitary-inter-renal axis of two trapping methods.

**Potential applications**

We hope the methods provide a framework that can be tailored to reliably measure waterborne corticosterone in different amphibian species across different contexts and environments including the field.

## Introduction

Ensuring animals experience good welfare is essential from an ethical standpoint but also to make sure animals are physiologically and psychologically healthy to serve as valid scientific or educational models ( Brod et al., 2019). The objective of this study was to validate the underpinnings of a method to non-invasively measure corticosterone, the amphibian stress hormone, from tank water. We have subsequently used these validated methods to refine housing and husbandry protocols to maximise welfare for captive *Xenopus laevis.*

Large numbers of amphibians are held in captivity for the purposes of research, conservation, education and as pets yet the field of amphibian welfare is a grossly neglected area partly due to the difficulty of interpreting amphibian behaviour ( Alworth & Harvey, 2007) and until recently ( Holmes et al., 2018) the lack of validated non-invasive tools for assessing stress physiology ( McClelland & Woodley, 2021). *X. laevis* (Daudin) is an important vertebrate model system in scientific research ( Wallingford et al., 2020). The world-wide research population is currently estimated at 52,000 ( Moss et al., 2024) and in Great Britain alone, Xenopus ( *X. laevis* and *X. tropicalis* combined) were the subjects of 15,003 procedures in 2023 (accounting for 0.6% of all scientific procedures, Home Office Statistics, 2023). Of these procedures 4,693 were for basic research, 7,178 were conducted for translational/applied research, 791 assessed protection of the natural environment and 2,341 involved the ‘creation and breeding of genetically altered animals not used in experimental procedures’ ( Home Office Statistics, 2023) [Data for 2023 were artificially high due to the inclusion of data omissions from the 2022 report which reports Xenopus were involved in 5,038 procedures (0.2% of all U.K. scientific procedures), Home Office Statistics, 2022]. Despite the large numbers of Xenopus in captivity and their use in scientific experiments there is minimal quantitative evidence pertaining to their management and welfare, especially in comparison to other model species such as primates, rodents and another aquatic species, the zebrafish *Danio rerio* ( Brod et al., 2019; Reed, 2005; Reed & Jennings, 2011; Tinsley, 2024). In comparison to Xenopus, empirical data informs aspects of husbandry for captive zebrafish such as diet ( Howells & Betts, 2009), enrichment requirements ( Delaney et al., 2002), social groupings ( Spence et al., 2008) and tank design ( Nema & Bhargava, 2016), possibly due to the ‘higher’ positioning of fish up the taxonomic ladder. Unlike amphibians, there are well-defined behavioural ethograms (e.g., Spence et al., 2008) and non-invasive methods to measure stress in the zebrafish ( Pavlidis et al., 2013).

Over the last five years our lab has been attempting to fill this knowledge gap for amphibians by developing and validating non-invasive behavioural and physiological tools to monitor and quantify amphibian welfare. Amphibians possess a hypothalamic-pituitary-inter-renal (HPI) axis (akin to the mammalian hypothalamic-pituitary-adrenal axis) which in addition to controlling physiological processes such as energy mobilization ( MacDougall-Shackleton et al., 2019), is activated under conditions of stress ( Narayan et al., 2019). Exposure to a stressor activates the amphibian HPI axis to release a suite of hormones, including corticosterone [CORT, a glucocorticoid (GC), which has a broadly equivalent role in amphibians to cortisol in other taxa]. CORT is the major GC in most ( Moore & Jessop, 2003), but not all amphibians ( Hopkins et al., 2020). For example, cortisol (rather than CORT) is the dominant GC in three species of Dendrobatid poison frogs (i.e., Anthony’s poison frog *Epipedobates anthonyi*, mimic poison frog *Ranitomeya imitator*, and Zimmermann’s poison frog *Ranitomeya variabilis* ( Westrick et al., 2023). We have shown CORT, rather than cortisol to be the dominant waterborne GC in *X. laevis* (Holmes et al., unpublished results). This is supported by in vitro work showing CORT (rather than cortisol) is released from isolated adrenocortical cells of *X. laevis* following stimulation ( Chan & Edwards, 1970). Hence our methods focussed on quantifying waterborne CORT as opposed to cortisol. CORT promotes changes at the molecular level e.g. working in tandem with the sympathetic nervous system to increase glucose availability and at the organismal level by altering behaviour such as feeding ( Maniam & Morris, 2012) that enables the animal to cope with the stressor and restore physiological homeostasis ( Wingfield & Romero, 2010). Chronically raised levels of GCs however implement a host of detrimental physiological changes including impaired immune function ( Webster et al., 2002), suppressed reproductive hormones ( Moore & Jessop, 2003), growth impairments ( Klasing, 1985) and altered cognition ( Smeets et al., 2009), together with behavioural changes such as suppressed sexual behaviour ( Moore & Miller, 1984). Although sometimes context dependent, there is a general consensus that GC levels correlate with survival probability ( Romero & Wikelski, 2001) and chronically raised GC levels compromise fitness ( Breuner et al., 2008; Vitousek et al., 2018). An understanding of a species’ stress response and the validation of tools to monitor HPI function are therefore imperative in order to measure and optimize welfare. In addition to regulating the stress response, CORT drives other, non-stress related functions in amphibians such as metamorphosis (by modulating the action of thyroid hormones, Denver et al., 2002), developmental rate ( Sterner et al., 2020) and the regulation of behavioural and physiological responses to pathogens ( O’Dwyer et al., 2024) all of which may benefit from our methods for the non-invasive measurement of CORT. We hope our methods have far reaching impact across a variety of scientific fields.

CORT is excreted into the holding water of amphibians although questions remain as to exactly how or from where. The holding water probably contains free CORT which has passed into the medium through the dermal layer ( Scheun et al., 2019) and from saliva ( Janin et al., 2012), together with metabolic products of CORT excreted in the urine ( Narayan, 2013) and faeces ( Szymanski et al., 2006) since GCs have been measured in all these media in amphibians.

Using techniques developed to measure levels of waterborne cortisol released by fish ( Scott & Ellis, 2007), we have developed and validated (both immunologically and biologically) non-invasive methods to extract and quantify levels of waterborne corticosterone released by Xenopus into a holding tank ( Holmes et al., 2016, 2018). The corticosterone probably originates from multiple sources as outlined in the paragraph above and indeed we have measured CORT in isolated faecal pellets from *X. laevis* (Holmes et al., unpublished results). We have robust immunological validations for specificity, accuracy, precision and sensitivity for our methods ( Holmes et al., 2018). Furthermore, our methods detect significant increases in waterborne CORT following presumed stressors such as transport, suggesting that waterborne CORT reliably reflects the HPI response ( Holmes et al., 2018). In most e.g., the tropical frog *Physalaemus pustulosus* ( Baugh et al., 2018; Gabor et al., 2013), but not all amphibian species e.g., spotted salamanders *Ambystoma maculatum* ( Millikin et al., 2019), waterborne CORT is correlated with plasma CORT providing physiological validation for the technique. The extent to which waterborne CORT correlates with whole body homogenates and/or tissue varies across species and developmental stage within a species ( McClelland & Woodley, 2021; Ruthsatz et al., 2023b) highlighting the complexities of linking chronic measures of CORT in the water with more transient levels in the body. For example, in the common frog *Rana temporaria*, waterborne CORT was correlated with tissue CORT in pro-metamorphic larvae but not newly hatched pre-metamorphic larvae, metamorphs or post metamorphic 10 day old froglets ( Ruthsatz et al., 2023b). This was most likely due to changes across developmental stages such as increased skin keratinization ( Nishikawa et al., 1989; Tata, 2006) and gill degeneration ( Burggren & West, 1982) which alters CORT passage across these routes ( Ruthsatz et al., 2023b). Although sometimes called for, we consider the robust biological validations obtained for our methods ( Holmes et al., 2016, 2018) refute the need for (and does not justify from an ethical standpoint) experiments assessing correlations of waterborne measures with plasma or tissue levels since our methods reliably detect increases in waterborne corticosterone during exposure to presumed stressors ( Holmes et al. 2016, 2018). Furthermore, several authors argue against the utility and validity of validating chronic, inter-grated measures of CORT present in amphibian waterborne samples with instantaneous, often pulsatile levels found in corresponding plasma or tissue. The latter is also more likely to reflect acute fluctuations due to biological and environmental events such as sample collection ( Millikin et al., 2019; Tornabene et al., 2021).

Methods used to measure HPI in amphibians traditionally involve venepuncture [ Heatley & Johnson, 2009; but see Jessica et al. (2024) for a minimally invasive method for collecting blood from the hind foot of amphibians via the *dorsalis pedis* vein using a 0.05 mm capillary tube], squeezing the whole frog for urine extraction i.e. by applying gentle pressure to the abdomen while holding the frog in the palm of the hand on its dorsal surface while the fingers and thumb are wrapped around the frog’s ventral surface to encourage urine expulsion ( Narayan, 2013), inserting oral saliva-collection devices ( Hammond et al., 2018), dermal swabbing ( Scheun et al., 2019), and whole-body homogenization ( Burraco et al., 2015). The latter are all invasive and in addition to causing potential harm, will confound HPI measurements to experimental treatments. Our methods for measuring HPI activity using waterborne CORT are non-invasive and involve placing a single frog in 1 litre of water for an hour and quantifying CORT released into the water. Our non-invasive method has several advantages over these invasive methods including: (1) it is non-invasive (and hence ethically more acceptable), (2) as it is non-invasive, HPI responses to experimental treatments are not masked by sampling effects as they would be for other CORT sampling methods such as venepuncture, (3) it is a suitable and valid method for obtaining samples from small aquatic animals (including many amphibian species) from whom it is challenging to obtain a blood, urine or saliva sample (e.g., spotted salamander larvae Millikin et al., 2019), (4) the method provides an integrated measure of GC that is not affected by internal pulsatile variation or external acute events ( Dickens & Romero, 2013), (5) animal sacrifice is not required as is necessary for whole body homogenate thus making it a more viable option for endangered species such as the related Lake Oku clawed frog *X. longpipes* ( Michaels et al., 2015), (6) repeated measures can be taken over an extended time frame without causing potential physiological challenges that repeated invasive methods may cause ( Gabor et al., 2013), (7) parallel behaviour measures can be taken at the time of hormone sampling which may be useful for interpreting GC levels ( Holmes et al., 2016), (8) since minimal handling is involved (compared to venepuncture, urine, saliva or dermal swab collection) our method reduces the risk of disease transmission between frogs – particularly relevant for field studies and the challenges posed with *Batrachochytrium* *dendrobatidis* (Bd) infection ( Gabor et al., 2015), (9) the reconstituted sample contains additional steroid hormones such as testosterone and progesterone which can be quantified ( Baugh et al., 2018; Ringler et al., 2024) and (10) several authors have shown levels of waterborne CORT to be highly repeatable across time ( Forsburg et al., 2019). One disadvantage of our method is it requires the frog to be singly housed for an hour which could raise ethical challenges in a social species. However, the experiments detailed below suggest the sample collection procedure does not cause significant or prolonged stress to the animal and the inclusion of robust control conditions can account for any potential confounds caused by the collection procedure should they occur.

Robust validation of hormone extraction techniques and assay methodology is essential for each new species and substrate ( Buchanan & Goldsmith, 2004; Palme, 2019) and this point has been discussed recently, specifically concerning waterborne CORT measures in amphibians ( McClelland & Woodley, 2021; Ruthsatz et al., 2023b). Methods to quantify waterborne CORT in amphibians are however in their infancy (e.g., Brod et al., 2019; Millikin et al., 2019). There are many unknowns for these latter methods pertaining to the most fundamental parameters such as the optimal type of sampling water and vessel (i.e., the water and vessel that do not compromise well-being and release minimal background interference compounds affecting the enzyme-immunoassay). The current paper reports a series of experiments elucidating essential factors that underpin the validity, reliability and robustness of methods to quantify levels of waterborne CORT for *X. laevis.* We have since used these validated methods (modified slightly and tailored for each species) to measure corticosterone and refine husbandry in a related species of conservation importance ( *X. longpipes*) in addition to the critically endangered Lake Zacapu Salamander, A *mbystoma andersoni* and three newt species ( *Triturus cristatus, Lissotriton vulgaris* and *L. helveticus*). We hope these experiments will provide templates that will be useful to scientists validating waterborne hormone quantification methods for other aquatic amphibian species.

The first series of experiments (A) assessed parameters linked to the performance of our biochemical extraction methods and sampling procedures. Since the methods used in hormone extraction and quantification affect the results ( Jewgenow et al., 2020) it is essential that these are fully validated, reported and ideally standardized. In particular we tested a series of hypotheses evaluating optimal water type, sampling vessel, filtration methods, potential CORT degradation over time and the impact of our sampling procedures on CORT levels i.e., frog transfer to and single housing in a sampling container. To our knowledge there is minimal if any published evaluation of the above parameters relating to quantifying waterborne CORT in amphibians.

Fundamental knowledge of a species’ biology is essential for designing experiments robustly, accurately and reliably, and, just as crucially, in the interpretation of the results. The second series of experiments (B) evaluated parameters linked to the biology of Xenopus including the influence of circadian rhythm, sex and snout-vent length (SVL) on waterborne CORT levels and the impact of single housing for 48 hours on body mass (a downstream indicator of ‘stress’; Breuner et al., 2013). In most mammals there exists an evolutionarily conserved organization of physiological and behavioural patterns into clear rhythmic patterns coinciding with ecological parameters – typically light and dark. In many species these cyclic patterns are regulated by GCs with high levels of GCs when the animals wake, declining across the active period to reach a nadir when the animals retire to rest ( Smith et al., 2015). It was therefore important to understand circadian variation in CORT release in *X. laevis* in order to plan time frames in which to collect samples and to interpret GC levels - especially as there is minimal data pertaining to GC cyclicity in amphibians. There is some evidence to suggest increased locomotory activity in Xenopus during the night compared to the light period ( Oishi et al., 2004) therefore we predicted raised CORT titres in the dark compared to the light period to support the increased energetic demands. However, several anurans adjust their behavioural and physiological cycles in response to changing environmental parameters. For example wild, but not captive cane toads *Rhinella marinus* exhibit peak CORT during the night coinciding with behavioural activity ( Jessop et al., 2014). Hence any circadian rhythm detected in our subjects, while useful for planning and interpreting experiments in our captive animals, cannot be generalised across the species residing in other habitats or light schedules ( Secondi et al., 2021).

Sex differences in baseline CORT are apparent in many mammalian species as a consequence of the different reproductive behavioural and physiological priorities of the two sexes. The fine details and patterning of the latter are typically driven by breeding activity since reproductive hormones influence GC levels in most species including amphibians ( Moore & Jessop, 2003). A knowledge of baseline CORT across the two sexes is important for planning experimental designs and interpreting CORT data, although not all studies assessing waterborne CORT report the sex of the subjects, often due to challenges with determining the sex of small amphibians or animals at certain developmental stages (e.g., Gabor et al., 2013). The majority of studies detect no sex differences in baseline CORT for amphibians using a variety of media including water samples ( Gabor et al., 2016) dermal secretions ( Scheun et al., 2019), urine ( Narayan et al., 2011) and saliva ( Hammond et al., 2018) hence we predicted equal levels of baseline waterborne CORT across the sexes.

There is debate in the literature on the scientific validity of controlling for body size when interpreting levels of waterborne GCs in amphibians despite many studies assessing waterborne CORT in amphibians doing so. This may be of particular relevance for species with sexually dimorphic body sizes such as *X. laevis* since females are significantly larger than males (the approximate weights of subjects in our laboratory are 70 g for males and 150 g for females). Some previous studies reporting CORT values in amphibians have controlled for body mass (spotted salamanders, Charbonnier et al., 2018) or SVL (spotted salamanders, Millikin et al., 2019) whereas others have no control for body size ( *X. laevis*, Holmes et al., 2018). Alternatively some studies report two sets of CORT values – one in which body size is controlled for and one which has no control for body size (e.g. Northern leopard frogs *Lithobates pipiens;* McClelland & Woodley, 2021). The type of correction factor employed can significantly impact relationships and patterns between waterborne CORT measures in addition to the absolute concentrations hence should be empirically validated to justify their use ( Millikin et al., 2019). Here we systematically test hypotheses relating to relationships between SVL and CORT in Xenopus to provide empirical evidence underpinning how we compute CORT levels in this species. Based on the paucity of literature documenting a relationship between SVL and CORT levels we predicted no relationships between SVL and CORT release in Xenopus.

## Methods

### General methods

***Subjects***

Subjects were wild-type adult *X. laevis*, purchased from the European Xenopus Resource Centre (EXRC, University of Portsmouth) in 2013 and subsequently housed at the University of Chester. Animals were included in experiments if they displayed no obvious signs of ill health and if they had not been used in an experiment for at least two weeks. Since all our experiments were non-invasive (i.e., did not require a home office license) we assumed that frogs experienced no long lasting harm following use – an observation supported by our work reported here in Experiments A5(i) and (ii) described below during which we showed that repeated transfer of a frog to a new tank did not raise levels of corticosterone over a 4 or 8 hr period. The individual ID of frogs used in each experiment are shown in Table 1. The weights of individuals at the start of each experiment are presented in the associated data file ( Smith et al., 2024a). There are more female than male frogs in our colony hence experiments testing biochemical methods utilised only female frogs as sex was irrelevant. This strategy ensured we had enough animals to perform timely experiments while giving subjects a rest period of two weeks between use in an experiment. Although there is no scientific evidence to justify the optimal rest period for amphibians, two weeks is a communally agreed time frame among Xenopus users (pers comm, Amphibian Welfare and Care Workshop, London June 27 ^th^ 2024). Frogs were immediately returned to their home tank at the end of each experiment.

**Table 1.** Individual identifiers (IDs) of animals used in each experiment. Weights of each individual at the start of the experiment are provided in the associated data set ( Smith et al., 2024a). A horizontal line signifies that animals of that sex were not used in the experiment.

Experiment	Female ID	Male ID
A1	-	-
A2	-	-
A3i	1, 3, 4, 5, 6, 7, 8, 9	-
A3ii	1, 2, 3, 4	-
A4	1, 3, 4, 5, 6, 7, 8, 9	-
A5i	2, 3, 6, 10	12, 13, 14, 15
A5ii	1, 2, 8, 10	11, 13, 14, 15
B1	1, 2, 3, 4, 5, 6, 7, 8, 9, 10	11, 12, 13, 14, 15
B2	1, 2, 3, 4, 5, 6, 7, 8, 9, 10	11, 12, 13, 14, 15

### Housing and husbandry of the frogs

Details on the housing and husbandry of the frogs are provided in Holmes et al. (2016). Briefly, the frogs were housed in single sex groups of five animals in dechlorinated mains water at a depth of 280 mm with a temperature range 20–23 °C (air temperature 23–25 °C). Frogs were in visual, auditory and olfactory contact with other frogs. Water quality was maintained by Hamburg Matten-style biological filtration and partial water changes (∼50%) three times a week. Water quality was checked weekly for pH (6.4–7.6), nitrates (<20 mg/l), nitrites (<5 mg/l) and ammonia (<0.5 mg/l) using a dipstick water testing kit (King British). Subjects were housed under a 12:12 light:dark cycle. The diet consisted of 2.3 mm Royale Horizon Trout Pellets (Skretting) *ad libitum* three times a week. Home tanks were provided with one piece of black PVC tubing (140 mm × 118 mm × 50 mm, Floplast) and two terracotta pots as environmental enrichment ( Chumet al.,2013; Reed, 2005). The housing water of the frogs varied from that in which the frogs were housed for sample collection (i.e., deionised water). There could be potential welfare implications of temporarily housing frogs in deionised water (e.g., osmotic impacts) and a different water type to that of their home environment. Although our published work and the experiments below do not find any behavioural or hormonal evidence of compromised welfare caused by our collection methods we advocate further research in the field. Researchers familiar to the behaviour of *X. laevis* monitored their behaviour on a daily ad hoc basis in case of adverse or unexpected behaviours but none were observed.

Frog tanks were cleaned three times a week (Monday, Wednesday, Friday by the same researcher that handled the frogs) which included removing large pieces of debris with a net, carefully scrubbing the walls with a clean cloth and replacing 50% of the tank water with dechlorinated mains water. The frogs remained in the tank during cleaning and care was made to cause minimal disturbance and not to touch the animals. All equipment involved in frog husbandry and experiments was cleaned with F10 SC Veterinary Disinfectant Cleaner (Animal Safe).

### Collection of excreted corticosterone from the tank water

In all studies CORT was extracted from the water and quantified using the methods and assay systems validated and described in Holmes et al. (2016, 2018). Briefly, individual subjects were singly placed in plastic tanks (length 210 mm × width 130 mm × height 140 mm) in 1 – 5 L deionized water (Labwater 1, Purite) for a period of time (the volume of water and time in the tank varied for each experiment as detailed below) and then always returned to their home tanks. All frog transferals between tanks were conducted by the same, trained researcher to reduce data variability. Frogs in our colony were used to being transferred between different tanks. The subject was calmly caught using two gloved (Powder-free vinyl gloves from Nutouch New Generation), cupped hands and immediately transferred as required. A net was not used for capture to avoid skin abrasion. The capture and transfer process took approximately 20 seconds. Gloves were worn throughout the sampling procedure and changed for the subsequent sample processing procedure. Gloves were not changed between each animal transfer process. Gloves were also changed at any point if they became contaminated for example with faeces.

The whole volume of tank water in the sample collection tank (unless otherwise specified in the experiments below) was vacuum filtered twice using a peristaltic pump (Gilson Minipuls3 Model 312) through filter paper with a pore size of 11 μm (Fisherbrand, Thermo Fisher Scientific) followed by cellulose nitrate filter paper (pore size = 0.45 μm, Sartorius). The total filtration procedure took between 45 minutes and 3 hours depending on the amount of debris i.e., typically shed skin, faeces and regurgitated food, in the water. The whole sample was pumped through activated solid phase extraction cartridges (previously primed with 5 ml HPLC-grade 100% methanol and 5 ml distilled water, Sep-pak Plus C18, Waters Ltd., U.K.) at a rate of 5 ml/min ( Ellis et al., 2004). Impurities were washed from the cartridges with 5 ml distilled water using the same rate of 5 ml/min ( Ellis et al., 2004) and the cartridges stored at - 20 °C until elution. GCs were eluted from the cartridges into borosilicate glass tubes (16 mm × 100 mm) (Fisherbrand, Thermo Fisher Scientific) using 4 ml ethyl acetate injected at a steady rate of 5 ml/min. The ethyl acetate was evaporated under nitrogen at 37 C using a Thermo Scientific™ Reacti-Vap 27 port Evaporator and Stuart Block Heater Module SBH1300/3. Samples were re-suspended in 500 μl EIA PBS buffer (0.1 g BSA in 100 ml 0.1 M PBS. PBS = 5.42 g NaH _2_PO _4_H _2_O, 8.66 g Na _2_HPO _4_ (anhydrous), 8.7 g NaCl, 1000 ml d-H _2_O, pH 7) and vortexed (Multi-Reax, Heidolph) at 1600 rpm.

### Experimental design

Sample size: Due to the novelty of our work there was no prior waterborne CORT data for *X. laevis* upon which to conduct power analysis to select sample sizes. Therefore, for each experiment we used the maximum number of adult frogs available from either one or both sexes that had been residing in control conditions, without experimental manipulation, for at least two weeks. As our work progressed, we observed large inter-individual variation in CORT levels as reported by others in the field ( Gabor et al., 2016; Tornabene et al., 2021) hence our strategy of using the maximum number of frogs continued throughout the validation studies. Furthermore, in all the experiments below every attempt was made to conduct repeated-measures experimental designs and process samples from a single frog, experiment, or phase of an experiment on the same day and in the same enzyme-immunoassay to reduce variability.

Blinding: ‘Blinding’ was not possible in our work due to our strategies to reduce sample variance explained above. Therefore, the researcher allocating frogs to experimental and control conditions was not blind to the conditions and a second researcher conducting the enzyme-immunoassays was not blind to the sample origin.

Randomisation: The order of sampling and allocation to control and experimental groups was determined by a researcher selecting samples at their free will hence systematic randomisation was not conducted.

Control groups: As animal control groups were not scientifically necessary to us achieving our objectives we did not include these in our experiments. However, we advocate the inclusion of control tanks i.e., tanks containing water but no frog, in all experiments to control for background levels of waterborne CORT (see also Ruthsatz et al., 2023b). This was not always possible however in the experiments reported here due to practical logistics and resource availability.

**Ethical considerations**

All the procedures were conducted in consultation with the UK Home Office, in accordance with the University of Chester Research Guidelines and under approval from the University of Chester Faculty Research Ethics Committee (FREC ref no 986/14/CH/BS 25, approved on the 25th November 2014).

We attempted to minimise disturbance and potential suffering to the animals as described in detail above. In summary (i) animal handling and cleaning was performed by the same researcher to minimise unpredictability, (ii) every attempt was made to cause minimal disturbance during cleaning, (iii) frogs were captured calmly using gloved hands (rather than a net which could cause skin abrasions), (iv) subjects were always returned to their home group immediately after use in an experiment, (v) group composition remained constant for the duration of the studies unless an animal had to be removed for health reasons (fostering social predictability) and (vi) the subjects were ‘rested’ for at least two weeks between use.

### Protocol for the Corticosterone enzyme-immunoassay

Corticosterone in samples was quantified using an enzyme-linked immunosorbent assay (ELISA), validated for quantifying CORT in water samples from *X. laevis* and described by Holmes et al. (2018). When possible, repeated measures samples from a single frog and samples from a single experiment were analysed on the same enzyme-immunoassay plate to reduce within frog and within experiment variation (32 samples could be run in duplicate on a single enzyme-immunoassay plate). In brief, 50 μl of antibody CJM006 (rabbit anti-corticosterone-3-CMO-BSA polyclonal antibody, produced by C. Munro, University of California, Davis, CA) at a 1 in 20,000 dilution in 0.05 M assay coating buffer (1.59 g Na _2_CO _3_, 2.93 g NaHCO _3_, 1000 ml d·H _2_O, pH 9.6), was used to coat plates which were incubated at 4 °C overnight. Plates were washed three times with ELISA wash buffer (40 g NaCl, 1 g KCl, 1.2 g KH _2_PO _4_, 7.2 g Na _2_HPO _4_, 1000 ml d·H _2_O, 1.5 ml Tween 20, pH 7.4). 50 μl of EIA PBS buffer (recipe above) were added to all wells, followed by 50 μl of corticosterone standard (C2505 Sigma-Aldrich) in duplicate (30,000 pg/ml – 58.6 pg/ml) or duplicates of 50 μl sample diluted 1:2 in EIA PBS buffer. A volume of 50 μl of corticosterone-hydrogen peroxidase conjugate (C. Munro, University of California, Davis, CA) at a 1 in 40,000 dilution in EIA PBS buffer was added to all wells and plates and incubated with gentle shaking at 100 rpm, at room temperature in the dark for 3 hours. Plates were washed as before and 100 μl per well of ABTS substrate [0.04 mM 2,2′-azino-di-(3-ethylbenzthiazoline sulfonic acid) diammonium salt (ABTS), 1.6 mM H _2_O _2_, 0.05 M citrate pH 4.0] was added. Plates were incubated at room temperature with shaking at 600 rpm for approximately 1 hour (MRX II Dynex Technologies) until the OD _405nM_ reached a value of 1. Plates were read and data computed using the program Revelation Version 4.22. Samples were re-assayed if the co-efficient of variation was >5%.

### Statistical evaluation

In all studies, the single unit of analysis was CORT concentration measured from a single tank, collected over a period of time defined in the experiment. The tank either contained a single frog, or no frog (i.e., a control tank that measured background levels of CORT). CORT rates are expressed as the cumulative amount of hormone excreted over the sampling period (which was typically an hour and is detailed for each experiment) and are expressed as pg/sample. A summary of the data is provided by the mean +/- standard deviation of the mean (SD). Data points were only excluded if levels of CORT exceeded those measured in the background tanks (containing no frogs) for a particular experiment and are detailed below in the individual experiments. All statistical analysis was carried out using GraphPad Prism 9.3.0. Statistical details for each experiment are detailed below. All analyses were two-tailed with a significance level of P < 0.05.

## Methods for individual experiments

### Experiments A: Biochemical aspects of the assay

***Experiment A1. Corticosterone levels in five different water types***

The goal of the experiment was to assess background CORT measured in five different water types (readily available in a laboratory setting) listed below. The water had not housed frogs at any time point hence this experiment was evaluating the extent to which compounds in the water and or leaching from the container interfere with the enzyme-immunoassay. Different water types might favour differential chemical leaching. Levels of background CORT may be particularly important when measuring waterborne CORT in small amphibians such as tadpoles who may absorb ambient CORT through the skin, thus confounding results ( Tornabene et al., 2021).

Hypothesis A1. There is no difference in CORT measured in 1 L aliquots of (1) standard tap water that has remained open to the air for de-chlorination in a 100 gallon plastic tub, (2) fresh tap water directly from the tap (3) distilled water, (4) deionized water (Labwater 1, Purite) and (5) ‘cleaned’- standard, dechlorinated water that has been filtered twice and passed through a primed Sep-Pak column as described above in the section ‘Collection of excreted corticosterone from the tank water’.

*A1 Methods*

No frogs were used in this experiment. For each of the five water types, four 1 L aliquots were placed in a plastic tank (210 mm × 130 mm × 140 mm) at ambient temperature for an hour upon which the sample was filtered and processed for CORT as described in the section ‘Collection of excreted corticosterone from the tank water’. A volume of 1 L was used since this was the volume of water used to house the frogs during sample collection. We were constrained to four aliquots for each water condition due to the availability of plastic tanks.

*A1 Statistical analysis*

Log transformed CORT levels were compared across the five water types using a between subject one factor ANOVA. Post hoc comparisons were made using Tukey Honest Significant Difference test comparing for multiple comparisons.

*A1 Results*

**Figure 1.**
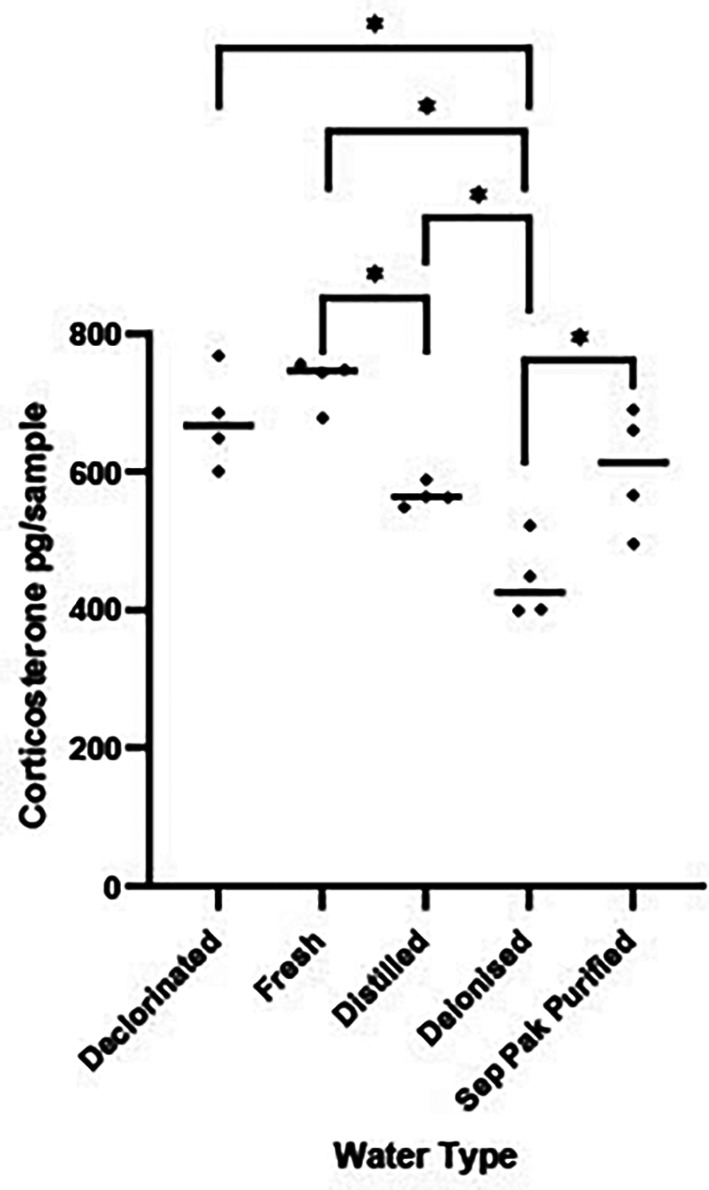
Background corticosterone levels in five water types. Scatterplot showing corticosterone (CORT) levels (pg/sample measured in 1 L water that was held in a tank for 1 hr) measured in five different types of water. The horizontal bar represents the median CORT pg per water type. Significant variations in log transformed CORT levels across the water types were determined using a between subject one factor ANOVA [F (4, 15) = 14.36, P < 0.0001]. Water types showing significant differences (P < 0.05) are denoted by *.

There were significant differences in the quantity of CORT measured in the five water types [F (4, 15) = 14.36, P < 0.0001, Figure 1]. Post hoc tests revealed deionised water had significantly less CORT than the other four water types (P < 0.02 for all comparisons). Distilled water had less CORT than fresh tap water (P < 0.05). On the basis of these results deionised water was used as the housing medium into which frogs were placed during CORT collection due to the comparatively low background levels of CORT.

***Experiment A2. Impact of vessel material on background levels of corticosterone***

The aim of the experiment was to evaluate background CORT measured from water held in glass or plastic sampling tanks.

Hypothesis A2. There is no difference in levels of background CORT between water held in glass or plastic tanks.

*A2 Methods*

A volume of 1 L de-ionized water was placed in six glass and six plastic tanks (210 mm × 130 mm × 140 mm) for 24 hrs without frogs. We used a 24-hr period to obtain a conservative estimate of CORT contamination from the vessel. Water was placed in alternating tank types on the shelf. At the end of the 24-hr period the samples were processed as described in the section; ‘Collection of excreted corticosterone from the tank water’.

*A2 Statistical analysis*

Log transformed CORT levels were compared across the two vessel types using an independent t test.

*A2 Results*

**Figure 2.**
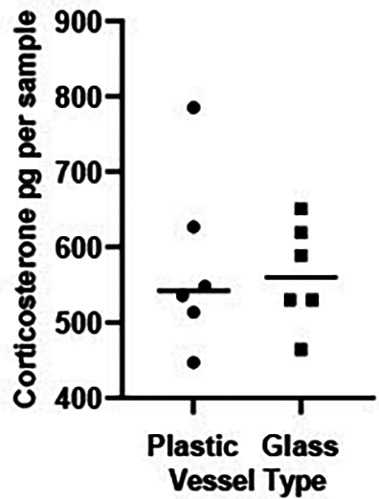
Background corticosterone levels in water sampled in plastic and glass tanks. Scatterplot showing corticosterone (CORT) levels (pg/sample measured in 1 L water held in a plastic or glass vessels for 24 hrs). The horizontal bar represents the median CORT per vessel. An independent t test on log transformed CORT values revealed levels of background CORT did not vary across vessel type (t = 0.216, df 10, P = 0.800).

There was no significant difference in levels of CORT measured in water held in glass versus plastic tanks (t = 0.216, df 10, P = 0.800, Figure 2).

***Experiment A3. Amount of ‘spiked’ corticosterone extracted from a water sample***

The goal of this experiment was to assess the impact of filtration on CORT levels recovered from the deionised water since methodological applications for GC analysis impact results ( Jewgenow et al., 2020).

Hypothesis A3. There is no difference in the amount of CORT recovered from samples spiked with CORT before versus after filtration.

*A3i Methods*

A total of eight female frogs were placed in individual experimental plastic tanks containing 5 L of water for 48hrs (see Table 1). 5 L was used to ensure a large enough volume from which multiple water aliquots could be taken. For the purpose of this experiment, two 500 ml aliquots were taken from each 5 L sample. One 500 ml aliquot was spiked with 1ml of 5000 pg/ml CORT (Cat No. C-106 1Ml, Merck UK) prior to filtering [made by adding 1000 μl of the corticosterone top standard (30,000 pg/ml) to 5000 μl EIA PBS]. The sample was filtered, passed through the sep pak column and levels of CORT quantified in the enzyme-immunoassay. The second 500 ml aliquot was unspiked, filtered, passed through the sep pak column and CORT levels quantified in the enzyme-immunoassay.

Water that had housed frogs was used in case animal components such as skin debris impacted filtration efficiency. The experiment used 500 ml water instead of the 1 L typically used when sampling frogs. Since we were spiking the 500 ml sample with 5000 pg/ml synthetic CORT we were confident of yielding adequate amounts of CORT to be detected in our assay. However, we were not confident of consistently obtaining enough CORT from a 500 ml frog sample to be detected by our assay, hence why we used 1 L water as standard frog sampling protocol in our subsequent experiments.

*A3i Statistical analysis*

The latter unspiked sample provided the baseline CORT titres from which the percent recovery of spiked CORT was computed. The percentage of the spiked 5000 pg/ml CORT recovered was computed by dividing CORT recovered i.e., CORT measured in spiked sample minus CORT measured in unspiked sample/CORT added multiplied by 100.

*A3ii methods*

A second experiment employed a similar experimental design to above except the first 500 ml aliquot was spiked after filtration and before passing through the sep pak. A total of four (and not eight) female frogs were placed in individual experimental plastic tanks containing 5 L of deionised water for 48 hrs (see Table 1). Two 500 ml aliquots were taken from each 5L sample as above. One 500 ml aliquot was spiked with 1ml of 5000 pg/ml CORT (Cat No. C-106 1Ml, Merck UK) after filtration and before passing through the sep pak [made by adding 1000 μl of the corticosterone top standard (30,000 pg/ml) to 5000 μl EIA PBS], passed through the sep pak column and titres of CORT quantified in the assay. The second 500 ml aliquot was unspiked, filtered, passed through the sep pak column and CORT titres quantified in the enzyme-immunoassay.

*A3ii Statistical analysis*

The latter unspiked sample provided the baseline CORT titres from which the amount and then percent recovery of spiked CORT was computed. The percentage of CORT recovered was computed as described above for Experiment A3i.

Frogs were present in all water samples to control for biological material originating from the frogs that could impact filtration efficiency. For example significant amounts of shed skin were usually present on the filter paper.

*A3 Results*

We obtained a mean +/- SD recovery of spiked CORT of 113.71 +/- 19.77 %, for samples that were spiked before the filter stage (A3i) and 111.12 +/-4.50% for samples that were spiked after filtration but before passing through the sep pak (A3ii). The results show that the filtration process does not remarkably alter levels of CORT recovered.

***Experiment A4. Assessing if corticosterone degrades in tank water over time***

Since amphibians are of extreme conservation importance ( IUCN, 2024), increasing number of studies are measuring HPI function in wild subjects in the field (e.g., Narayan et al., 2019; Ruthsatz et al., 2023b). There are several challenges associated with collecting samples from field settings such as whether to use locally or remotely sourced water ( Gabor et al., 2018), transporting large volumes of sampled water and a time delay in loading collected samples on the sep pak cartridge and transfer to – 20 °C. Experiment 4 evaluated potential degradation of CORT over time in the sampling water that had held frogs. Even though our standard protocol holds frogs for 1 hr in 1 L water, in this experiment we held frogs for a 48-hr period in 5 L water to ensure adequate amounts of CORT and sampling water to achieve the study objectives. This experiment is of relevance to ascertain if CORT degrades over time in samples for which there is a delay to process as may happen to samples collected in remote field sites.

Hypothesis A4. CORT measured in a single water sample (that had housed a frog) does not change over a 48-hr period.

*A4 Methods*

A total of eight female frogs were placed in individual experimental plastic tanks containing 5 L of deionised water for 48 hrs (see Table 1). A volume of 5 L was used so multiple water measurements could be taken over the 48-hr period. A volume of 500 ml of water was removed for sampling at each timepoint as follows. After frog removal at 0900 hrs the housing water was sampled at 6 time points: 0 hrs (0900 hrs), +2 hrs (1100 hrs), +4 hrs (1300 hrs), +8 hrs (1700 hrs), +24 hrs (0900 hrs) and +48 hrs (0900 hrs). The samples were processed as described in the section ‘Collection of excreted corticosterone from the tank water’ and levels of CORT measured in the enzyme-immunoassay.

*A4 Statistical analysis*

Log transformed CORT concentrations were compared across the six time points [0 hrs (0900 hrs), +2 hrs (1100 hrs), +4 hrs (1300 hrs), +8 hrs (1700 hrs), +24 hrs (0900 hrs) and +48 hrs (0900 hrs)] using a repeated measures one factor ANOVA. Post hoc comparisons were made using Tukey Honest Significant Difference test corrected for multiple comparisons.

*A4 Results*

**Figure 3.**
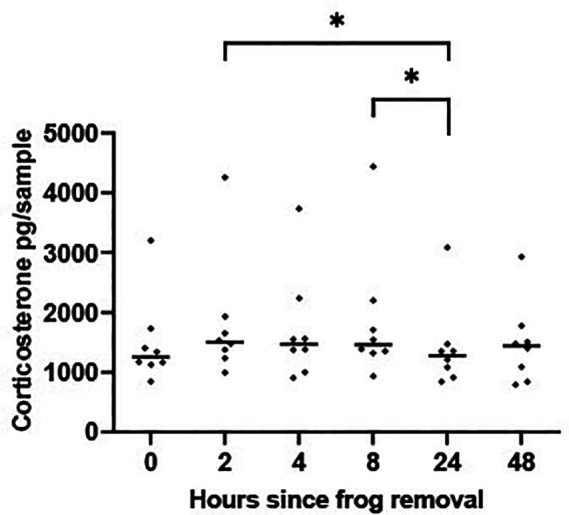
Corticosterone levels in a sample held at ambient temperature change over 48 hours. Scatterplot showing corticosterone (CORT) levels (pg/per sample measured in sequential 500 ml water aliquots collected at six time points from a 5 L sample that had housed a frog for 48 hrs). The horizontal line represents the median CORT value. A mixed-effects ANOVA determined that log transformed waterborne CORT levels changed significantly across the 6 time periods [F (5, 35) = 3.872 P = 0.007]. Time periods showing significant differences (P < 0.05) are denoted by * and were determined using Tukey Honest Significant Difference test corrected for multiple comparisons.

There was a significant difference in CORT levels measured in the experimental tanks across the six points suggesting that concentrations of water CORT change in a sample kept at room temperature over a 48 hr period [F (5, 35) = 3.872 P = 0.007], Figure 3. Pairwise comparisons showed that concentrations of CORT in the water at 2 hrs and 8 hrs were significantly higher than those at 24 hrs (P < 0.05). CORT measured at 48 hrs did not vary significantly from CORT measured at any other time points.

***Experiment A5. Impact of sampling procedure on frog corticosterone levels***

The goal of the study was to assess the impact of repeated sampling on frogs – i.e., repeated handling and transfer between tanks especially since handling has been shown to raise waterborne CORT in amphibians ( Gabor et al., 2016). This would further our understanding of the impact of our methods on frog HPI function and would establish if our method was stressful and if it confounds CORT response to experimental treatments. As such, this was important for ethical reasons and to interpret our CORT results to experimental treatments. The first experiment (A5i) transferred four male and four female frogs to a new sampling container every 30 minutes spanning a four-hour period. A 30-minute sampling period was selected to assess if there was an initial HPI response to the handling/transfer procedure which could be diluted (and potentially missed) if CORT concentrations were only assessed after an hour. The second experiment (A5ii) evaluated the impact of our sampling methods over an extended time period; transferring four male and four female frogs every 60 minutes for an 8-hour period. The 60-minute period was used to mimic experimental sample collection procedures.

Hypothesis A5i. Repeated handling and transfer of frogs between containers every 30 minutes does not affect CORT levels over a 4-hour period.

Hypothesis A5ii. Repeated handling and transfer of frogs between containers every 60 minutes does not affect CORT levels over an 8-hour period.

*A5 Methods*

(i) 30 minute handling and transfer: Individual frogs (four males and four females; Table 1) were placed in plastic tanks (dimensions length 254 mm × width 203 mm × height 208 mm) containing 1 L deionized water at 0900 hrs. After 30 minutes each individual frog was moved to a clean tank (containing 1 L deionized water freshly placed in the tank). This process was repeated a further six times yielding eight samples per frog. The experiment spanned four days with a one male and one female being manipulated each day. At the end of the 4 hour period levels of CORT were measured in the eight samples using methods outlined in the section ‘Collection of excreted corticosterone from the tank water’. Two control tanks were set up each of the four days with 1 L of water which were all sampled at the end of the 4-hr period. Mean background CORT computed from the two control tanks on each day was deducted from all samples collected the same day.

ii) 60 minute handling and transfer: The study employed four male and four female frogs (see Table 1). The experimental design was the same as above (A5i) but individual frogs were transferred to a new container containing fresh deionised water every 60 minutes over the course of 8 hours (i.e. six transfer procedures yielding eight samples per frog). Additionally, two males and two females were run on each day (i.e. the experiment lasted 2 days). At the end of the 8 hour period levels of CORT were measured in the eight samples using methods outlined in the section ‘Collection of excreted corticosterone from the tank water’. Two control tanks were set up each of the two days with 1 L of water. All control tanks were sampled at the end of the 8-hr period and mean CORT computed from the two control tanks on each day was deducted from all samples collected the same day.

*A5i and ii Statistical analysis*

Log transformed CORT concentrations were compared across the samples from the eight time periods each from study using mixed-effects ANOVAs to accommodate missing data points. In Experiment A5i 11 samples had CORT concentrations that were lower than the concentrations of CORT in the empty control tanks (each subject had between 1 and 4 samples that were lower than those of the control samples). These 11 samples were removed from the analysis. In experiment A5ii where subjects were transferred between tanks hourly only 1 sample (after the 6 ^th^ hour) had CORT levels that were lower than those in the control tanks. The latter data point was removed. Post hoc comparisons were made using Tukey Honest Significant Difference *test corrected for multiple comparisons.*

*A5i and ii Results*

**Figure 4.**
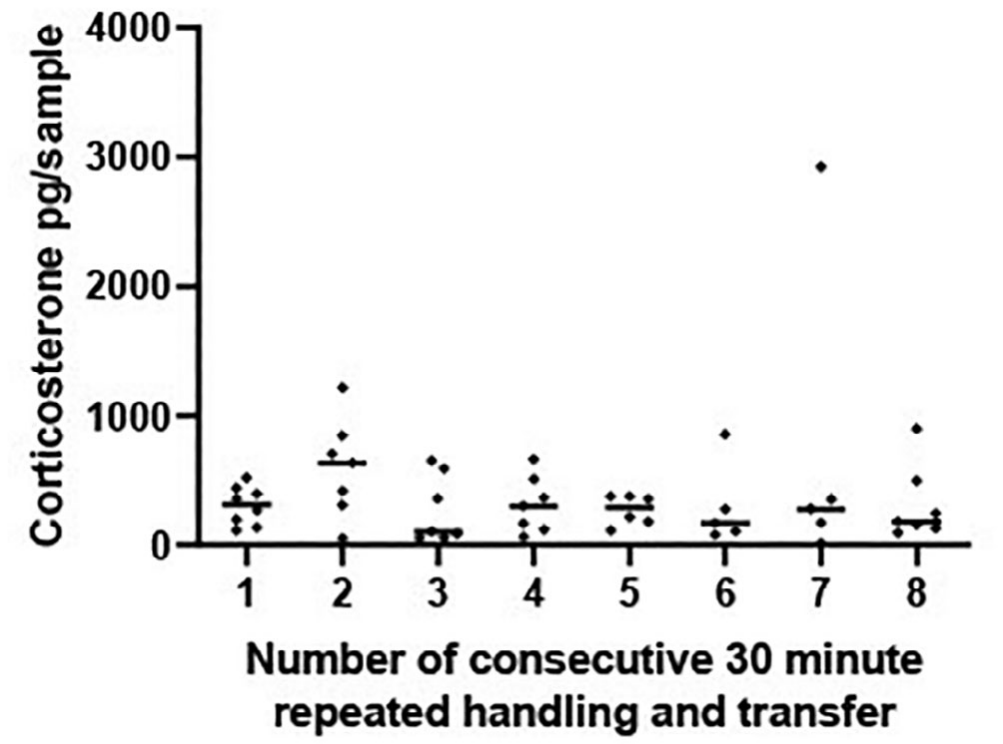
Corticosterone does not change with repeated transfer to a new tank over 4 hours. Scatterplot showing corticosterone (CORT) levels (pg/sample measured in 1 L water that had held a frog for 30 mins) from frogs experiencing eight repeated handling and transfer procedures to a new container containing 1 L of water. Frogs experienced one transfer and handling procedure every 30 minutes for a 4 hour period. The horizontal line represents the median CORT concentration at each 30 minute time period. A mixed effects ANOVA determined that concentrations of log transformed CORT did not vary significantly across the 8, transfer and handling procedures [F (7, 44) = 0.517, P = 0.816].

There was no significant change in CORT levels across the eight 30-minute time periods [F (7, 44) = 0.517, P = 0.816], Figure 4.

**Figure 5.**
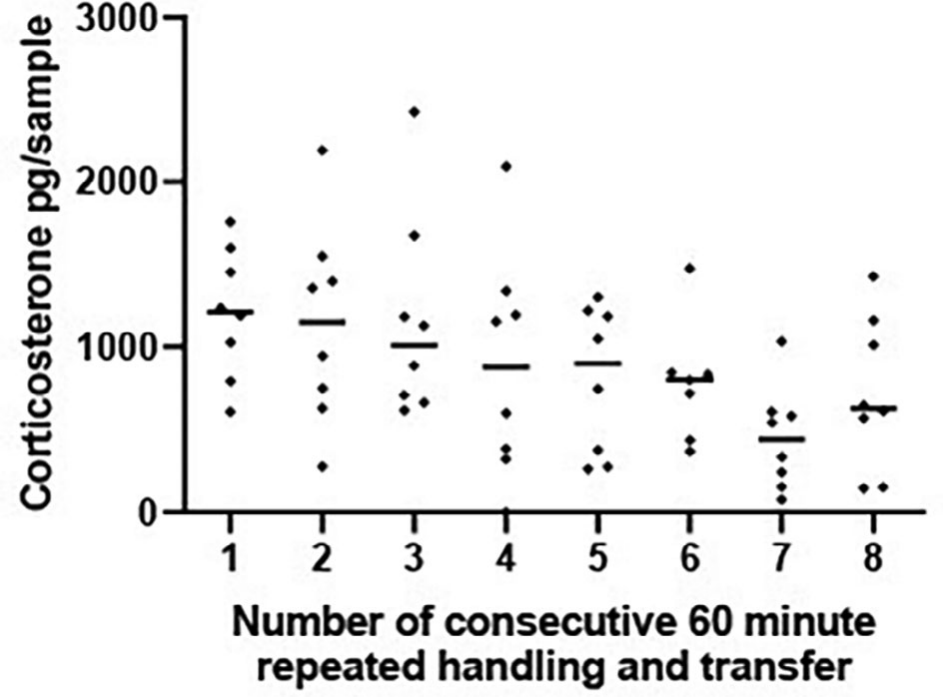
Corticosterone does not change with repeated transfer to a new tank over 8 hours. Scatterplot showing corticosterone (CORT) levels (pg/sample measured in 1 L water that had held a frog for 1 hr) from frogs experiencing 8 repeated handling and transfer procedures to a new container containing 1 L water. Each frog experienced one handling and transfer procedure every 60 minutes for an 8 hour period and remained in each tank for a 1 hr period. The horizontal line represents the median CORT concentration at each 60 minute time period. A mixed effects ANOVA on log transformed CORT concentrations determined that CORT did not vary significantly across the 8 handling and transfer procedures [F (7, 48) = 1.875 P = 0.100].

Similarly, when subjects were transferred between tanks hourly (experiment A5ii) CORT levels did not change significantly over the 8 hr sampling period [F (7, 48) = 1.875 P = 0.100, Figure 5].

### Experiments B. Impact of the biological parameters of Xenopus on levels of waterborne corticosterone

***Experiment B1. Circadian variation and sex differences in corticosterone***

Understanding circadian changes in levels of CORT, in addition to baseline sex differences in CORT is essential to plan time frames in which to conduct experiments and interpret CORT results. The study investigated variations in the amount of CORT released by 10 females and five males frogs during the total day (light) and night (dark) periods since circadian variation in plasma corticosterone has been reported in Xenopus ( Thurmond et al., 1986).

Hypothesis B1i. Levels of excreted CORT do not vary between the light and dark period

Hypothesis B1ii. Males and females have similar CORT levels

*B1 Methods*

Frogs were housed in an artificially imposed 12:12 light:dark cycle – with the lights coming on at 0800 hrs and off at 2000 hrs as is their normal housing arrangement. A total of 10 females and 5 males ( Table 1) were individually placed in 5 L of deionised water, for 12 hours either at the start of the light (0800 hrs) or dark (2000 hrs) phase. Using a repeated design the amount of excreted waterborne CORT was measured for each individual during 12 hours in the dark and 12 hours in the light with the two conditions being separated by one week. A total of five frogs had samples taken during the light period first, and 10 frogs had samples collected during the dark treatment first. 1 L of water was filtered from each tank immediately at the end of the light and dark phase as dictated by the experimental design and processed to quantify concentrations of CORT released during the preceding 12 hr light or dark period.

*B1 Statistical analysis*

Log transformed CORT titres were compared across the light and dark phase (repeated measure) and between the sexes using a mixed 2-factor ANOVA using log transformed CORT levels.

*B1 Results*

**Figure 6.**
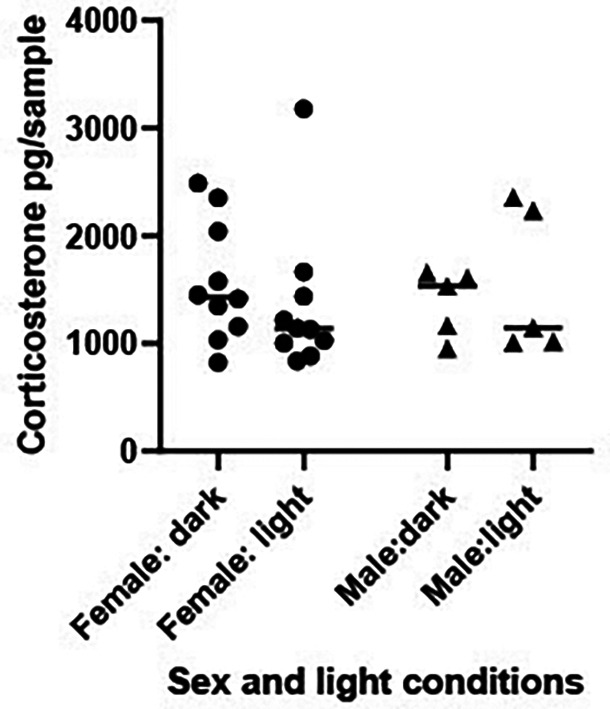
Corticosterone levels are comparable between males and females and during the day and night periods. Scatterplot showing individual corticosterone (CORT) (pg/sample measured from 1 L water taken from a 5 L volume which had held the frog for 12 hrs) levels for 10 females and 5 males measured over a 12 hour light and a 12 hour dark period. The horizontal line represents the median CORT value. A mixed 2-factor ANOVA determined there were no significant differences in cortisol levels between the sexes [F (1, 13) = 0.712, P = 0.414], or across the light and dark phases [F (1, 13) = 0.154, P = 0.701] or interaction between the two variables [F (1, 13) = 0.712, P = 0.414].

There was no significant difference in levels of CORT released in the light versus dark phase [HB1i: F (1, 13) = 0.154, P = 0.701, Figure 6] nor was there any effect of sex [HB1ii: F (1, 13) = 0.031, P = 0.863] or interaction between phase and sex [F (1, 13) = 0.712, P = 0.414] on CORT levels.

***Experiment B2. Impact of snout-vent length on CORT excretion***

Waterborne CORT levels are often controlled for body size (e.g., Millikin et al., 2019). We have shown previously that there was no correlation between CORT and *X. laevis* body mass for either sex (Pearson’s correlations: 10 females: r = 0.12, P = 0.780; 8 males: r = 0.40, P = 0.330, Holmes et al., 2018). The current experiment B2 explored whether SVL correlated with the amount of CORT produced and hence whether CORT values should be corrected for body size.

Hypothesis HB2. There is no relationship between SVL and the quantity of CORT excreted.

*B2 Methods*

Ten females and five males were individually housed in 5 L of tank deionised water for 48 hrs after which 1 L was removed for filtering and measuring CORT as described in the section ‘Collection of excreted corticosterone from the tank water’ (see Table 1). Frogs were weighed and their SVL measured immediately before housing in the tank. A soft tape measure was used to measure SVL by one researcher along a horizontal line while the frog was gently held by a member of the animal care staff using gloved hands.

*B2 Statistical analysis*

Spearman’s correlation was used to assess any potential relationship between SVL and CORT for each subject in males and females separately.

*B2 Results*

**Figure 7.**
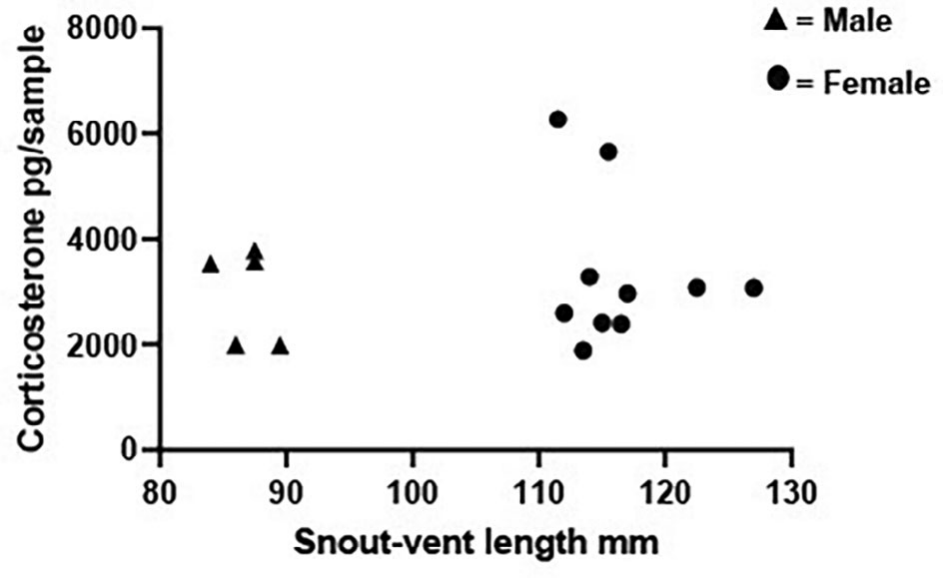
No relationship between snout-vent length and corticosterone levels. Scatterplot showing individual snout-vent length plotted against the individual’s corresponding corticosterone (CORT) levels (pg/sample measured from 1 L water taken from a 5 L volume which had held the frog for 48 hrs). Data are shown for 10 females and 5 males. Spearman correlations determined there was no relationship between snout-vent length and CORT for either males (r = -0.154, P = 0.833) or females (r = -0.042, P = 0.900).

There was no significant correlation between SVL and CORT levels in males (r = -0.154 P = 0.833) or females (r = -0.042, P = 0.900; Figure 7).

***Experiment B3***

Considering the challenges of interpreting GC levels, several authors advocate combining GC measures with other indicators of stress such as behaviour, body condition, telomere length, immune function or body mass among many (see Breuner et al., 2013). Body mass is a non-invasive downstream indicator of stress in mammals and amphibians ( Breuner et al., 2008, 2013) since weight loss is predicted to occur under conditions of stress due to lipolysis ( McClelland, 2004) and gluconeogenesis ( Breuner et al., 2013). It was important to see if our sampling methods reduced body mass in frogs. Although our sampling method requires only 1 hr single housing, we used 48 hr single housing to obtain a conservative measure of body mass change and to ensure a long enough period for any physiological consequences of single housing on body mass to be measurable.

Hypothesis HB3. Housing frogs in a sampling tank for 48 hrs does not affect body mass.

*B3 Methods*

Ten females and five males were individually housed in 5 L of tank deionised water for 48 hrs. Frogs were weighed immediately before and after housing in the tank. After frog removal, 1 L of the 5 L volume was removed for measuring CORT as described in the section ‘Collection of excreted corticosterone from the tank water’.

*B3 Statistical analysis*

Body weight was compared for males and females immediately before and after housing in 5 L of water for 48 hrs using a dependent t test.

*B3 Results*

Body mass did not change over the 48-hr sampling period for either males (t = 0.087, df 4, P = 0.935; mean +/- SD weight upon entry was 70.51 +/- 5.38 g versus 70.42 +/- 2.27 g 48 hrs later) or females (t = - 0.994, df 9, P = 0.346; mean +/- SD weight upon entry was 160.132 +/- 11.18 g versus 160.84 +/- 12.78 g 48 hrs later).

## Discussion

There is a critical need for the development and validation of non-invasive techniques for monitoring HPI function in amphibians ( Brod et al., 2019; Ruthsatz et al., 2023b). Here we report the validation of essential methodological and biological parameters to promote robust, reliable quantification of waterborne CORT in Xenopus and facilitate interpretation of CORT values which are inherently hard to interpret. Our non-invasive methods provide a refinement of current, invasive methods available for measuring HPI function in *X. laevis.* Our methods probably cause less stress than invasive methods and results presented here suggest they do not increase HPI function, do not reduce body mass (a downstream indicator of ‘stress’; Breuner et al., 2013) over a 48-hour period at least and our previous studies show they do not mask CORT increases to experimental treatments ( Holmes et al., 2016, 2018). By adapting and validating our methods for measuring HPI function to other species, researchers can fine tune protocols for housing and managing amphibians to optimise welfare. Animals with good welfare provide more robust, repeatable scientific data than animals with poor welfare ( Poole, 1997). Data variability is lower in this former group hence the number of animals required for scientific experimentation is reduced. Furthermore, animals with good welfare live longer and need to be replaced less often.

To our knowledge this is the first study to provide empirical data supporting choice of sampling tank, or water type when measuring waterborne CORT. We have shown that different water types harbour different amounts of background CORT which should be controlled for when measuring waterborne CORT and ideally standardised across laboratories and experiments. Ideally samples should be collected and transported in a water type and vessel that are ethically acceptable and have repeatable and low levels of background CORT to avoid masking experimental treatments. Our results highlight that deionised followed by distilled water harbour the least background CORT, hence our lab reliably uses deionised water as a sample collection media both in the laboratory and field settings ( Holmes et al., 2016, 2018). Chemical composition of the home-tank water for our captive animals i.e. dechlorinated tap water, is not too dissimilar to deionised water and therefore temporary housing in this medium for sample collection should not be stressful. Details on water types used to sample waterborne CORT in amphibians are often vague e.g. defined as ‘well’ or ‘spring’ water; ( Gabor et al., 2015) however dechlorinated water is a common media used in these type of studies ( Gabor et al., 2013). Deionised water may not be suitable however when sampling from wild animals whose native water quality will differ to that of captive subjects. Previous attempts to collect CORT samples in ‘natural’ sources such as local stream water have resulted in challenges ( Gabor et al., 2018). A high variance in background CORT levels was found in samples of local stream water used to sample Jollyville Plateau salamanders *Eurycea tonkawae* with higher values quantified in urban compared to rural environments: in some cases, background CORT was higher in control samples (i.e. containing no animal) than experimental samples ( Gabor et al., 2018). Similarly, high levels and variance in background CORT masked and confounded experimental response to salinity gradients in three amphibian species in a field setting (boreal chorus frogs *Pseudacris maculata*, northern leopard frogs *Rana pipiens* and barred tiger salamanders *A. mavortium*; Tornabene et al., 2021). It has been suggested that amphibians housed in water with high ambient CORT may absorb exogenous CORT through the skin, which downregulates the HPI axis and reduces levels of circulating CORT ( Gabor et al., 2018; Tornabene et al., 2021). Challenges associated with sampling CORT from water in a field setting suggests that water used to collect CORT from wild animals in situ, may need to be transported from the laboratory. This imposes limits on how remote an area can be studied due to the practical logistics of carrying water. We have successfully measured CORT in three species of wild newts ( *Triturus cristatus*, *Lissotriton vulgaris* and *L. helveticus*) in the Highlands of Scotland ( Miró, O’Brien et al., 2017) using deionised water transported from the laboratory and carried 2 km distance on foot between our vehicle and the field site showing our method is feasible in the wild.

The experiments reported here detected significant differences in background CORT across different water types highlighting the need for more research to ascertain optimal sampling water types for both laboratory and field settings that take into account not only levels of background CORT but the animals’ physiological osmoregulatory parameters – possibly using chemical buffers ( Baugh et al., 2018) or passing natural water through charcoal filters to remove confounding background CORT. Despite housing our subjects in water that differs chemically to their home tank water we do not detect an increase in HPI function in response to extended housing in deionised water (i.e. up to 8 hours housing in deionised water), nor do we observe reductions in body mass (a down-stream indicator of stress, Breuner et al., 2013) when animals are singly housed in deionised water for 48 hours suggesting the sampling conditions are not stressful over an extended period of time. This is in line with the natural ecology of *X. laevis* who will experience fluctuations in water quality in line with changing rainfall. In a recent study we sampled CORT from frogs housed singly for an hour in aliquots of their standard housing water taken from a Techniplast Xenopus recirculating water tank system and obtained immunologically and biologically relevant CORT results ( Moss et al., 2024). We advocate further research into optimal sampling water since Moss et al. (2024) show that alternatives such as home tank water are possible.

Repeated sampling i.e. handling and transferal to a new container, every 30 minutes, did not increase CORT values over a four hour period despite some previous studies showing handling increases waterborne CORT in amphibians ( Gabor et al., 2016). Equally, hourly transfer to a different container did not increase CORT over an 8-hour period providing empirical evidence that our sampling methods do not confound CORT results. Taken together these data suggest our sampling procedures are not acutely or cumulatively stressful and support the non-invasive nature of our procedure. Our methods made every attempt to reduce handling stress by restricting handling to one researcher, using cupped hands rather than a net which could cause abrasions on the skin and minimising the handling time. Similarly, Gabor et al. (2013) found no increase in waterborne CORT in *A. obstetricans* following repeated handling and transfer to a new container every 30 mins for 2 hrs. In our study there was a non-significant trend towards reduced CORT levels after 5 hours of hourly transfer – possibly due to a circadian decrease in CORT (excreted CORT decreases across the day in many media in mammalian species e.g. Smith et al., 2015) or habituation to repeated handling and transfer between sampling vessels ( Cyr & Romero, 2009). Future experiments should measure waterborne CORT at single 1-hour periods across the day to explore circadian variation in CORT release across the daylight hours. If our sampling procedure does initially stimulate HPI function although raising ethical concerns, this can be controlled for by using carefully designed experiments that include robust control conditions that control for methodologically induced HPI increases ( Holmes et al., 2018). Frogs mount a substantial CORT increase to experimental treatments such as transport up and above the baseline control levels (i.e. over 100% increase in GC levels) suggesting our sampling procedures per se have minimal if any impact on HPI function: GC levels after an initial 1 hour of sampling in Experiment 5ii were only around 25% higher than at the end of the day after seven frog transfers to a new tank ( Holmes et al., 2016, 2018). Similarly, baseline levels of CORT released from *Rana berlandieri* tadpoles held in a beaker over an hour period were significantly lower than CORT rates caused by adrenocorticotropic hormone (ACTH) challenge (i.e. injection with ACTH to stimulate the release of CORT from the interstitial tissue) and agitation, further supporting the results that temporary single housing for an hour does not confound the CORT response to experimental treatments in amphibians ( Forsburg et al., 2019). Finally, our results that single housing for 48 hrs in deionised water did not impact body mass (a down-stream indicator of stress in amphibians; Breuner et al., 2008; Millikin et al., 2019) supports our results that short-term single housing for an hour, a component of our sampling regime, is not detrimental to well-being. Systematic studies from the fish literature suggest the sampling procedure for waterborne CORT is only stressful when small bodies of water are used relative to the size of the fish ( Wong et al., 2008). Assessing the impact of tank size and water volume on sampling procedures in aquatic amphibians remains an important area for future research.

An optimal sampling period for waterborne CORT needs to be a trade-off between minimizing isolation time in an unfamiliar vessel yet long enough for meaningful amounts of CORT to be diffused into the water. Too long a sampling period will not only be unethical but could reduce the validity of the methods if spikes in CORT concentrations are dampened by extended collection periods, or if sampling conditions promote HPI activity and mask the response to experimental treatments. We used a one-hour sampling period in our studies since this yielded biologically relevant quantities of CORT in our species. The majority of studies measuring waterborne CORT across the amphibian group also use a 1 hr sampling period e.g. red spotted newts *Notophthalmus viridescens* ( Aspbury et al., 2017), the túngara frog *Physalaemus* *pustulosus* ( Baugh et al., 2018), Jollyville Plateau salamanders ( Gabor et al., 2018), larval spotted salamanders *Ambystoma maculatum* ( Millikin et al., 2019), larval and metamorphic Northern Leopard Frogs *Lithobates pipiens* ( McClelland & Woodley, 2021) and various developmental stages of the common frog *Rana temporaria* ( Ruthsatz et al., 2023b). Pharmacological justification for an hour sampling period was provided by Baugh et al. (2018) who showed that raised levels of plasma CORT following an ACTH challenge (and brief handling) in túngara frogs are reflected in waterborne CORT within an hour. In our current study a shorter sampling period of 30 minutes did not consistently collect biologically meaningful amounts of CORT since 11 or the 64 frog samples had levels of CORT lower than background tanks containing no frogs. Furthermore a 30-minute sampling period was associated with large variance in waterborne CORT levels both within and between individual frog samples (although overall levels of CORT were not statistically different across the eight 30 minute repeated sampling periods). Large inter-sample variation in waterborne CORT is widely reported and appears to be an inherent challenge in measuring waterborne CORT levels ( Tornabene et al., 2021; Gabor et al., 2016). Problems of large sample variance could be mitigated by increasing sample number ( Ruthsatz et al., 2023b) although this may have practical, time, cost and ethical implications. Sample size in any experiment should be carefully ascertained though the use of power calculations to ensure optimal animal use such that there are adequate numbers to offset high variability but not excessive animal recruitment as per the goal of the 3Rs to reduce animal usage (and avoid small effects becoming statistically significant). In our laboratory additional measures taken to reduce data variability include using repeated measures designs, restricting all animal transferral to and from the collection tank to one person, processing samples from a single frog, experiment, or phase of an experiment on the same day (if possible) and analysing repeated measures samples, from a single experiment on the same enzyme-immunoassay plate (if possible).

The concentration of CORT in a water sample left at room temperature fluctuated up and down over a 48-hr period. Here, CORT increased over 8 hrs and then returned to the initial value measured at time zero after 48 hrs, (in a water sample that had held frogs for 48 hours and was left at room temperature). Fluctuating levels of GCs in samples left at ambient temperature compared to immediate freezing are commonly reported in the literature (see Carbillet et al., 2023). Studies report increases in concentrations e.g., white-tailed deer *Odocoileus virginianus* and elk *Cervus elaphus* ( Millspaugh et al., 2003), orangutans *Pongo pygmaeus morio* ( Muehlenbein et al., 2012), cows *Bos taurus*, horses *Equus caballus* and pigs *Sus scrofa domesticus* ( Messmann et al., 1999); decreasing levels e.g., gorillas *Gorilla gorilla gorilla* ( Shutt et al., 2012) and crested macaques *Macaca nigra* ( Gholib et al., 2018) or no changes in GC concentrations when samples are left at ambient temperature prior to freezing ( Nugraha et al., 2017). Changing GC levels in both faecal and our water samples are probably caused by microbial and bacterial action degrading and altering CORT metabolites and releasing free CORT ( Hadinger et al., 2015). However, the direction of change is probably impacted by the type of microbial populations (which will change across environments, species and probably developmental stages; Messmann et al., 1999) and the analytical assay used to quantify CORT – specifically the immunoreactivity of the primary antibodies used in the enzyme-immunoassay ( Lexen et al., 2008). Our enzyme-immunoassay used a broad spectrum polyclonal antibody, (CJM006) which detects a range of corticosterone metabolites in addition to corticosterone which might explain the increase in CORT detected in our water samples left at ambient temperature ( Watson et al., 2013). Our results suggest water samples should be processed as soon as possible after collection and if this is not possible, the time to water processing should be standardised across an experiment.

There is a complex interplay between the HPI and Hypothalamic-pituitary-gonadal (HPG) axis in amphibians as with mammals ( Moore & Jessop, 2003). As expected, however, we found no differences in baseline levels of CORT across the two sexes. We have shown previously that body size and CORT are not correlated in *X. laevis* hence sexually dimorphic body sizes i.e. females tend to be larger, should not be confounding the results. However, it should be noted that since our statistical methods were testing for a difference, we cannot unequivocally state that CORT levels are equivalent between the sexes. No evidence was found for a sex difference in CORT for other amphibians including from water samples of *E. nana* and *E. sosorum* ( Gabor et al., 2016), dermal secretions of the bull frog *Pyxicephalus edulis* ( Scheun et al., 2019), urine from the cane toad *Rhinella marina* ( Narayan et al., 2011), or saliva of the American bullfrog *R. catesbeiana* ( Hammond et al., 2018) although concentrations of urinary CORT were higher in female than male Fijian ground frogs *Platymantis vitiana* ( Narayan et al., 2012). Although there is minimal evidence for sex differences in baseline CORT across amphibians several studies report marked sex differences in response to experimental treatments (e.g. Holmes et al., 2018) hence we still advocate the inclusion of sex as a variable in experimental design and data analysis. For example in Xenopus the patterning of the CORT response to transport varies across the sexes i.e. the rate at which CORT increases and decreases, although there is no difference in the magnitude of the CORT response across the experimental period ( Holmes et al., 2018). In contrast, in green frogs, *R. esculanta,* there are no sex differences in the initial trajectory of CORT increases following capture but the magnitude of the CORT response varies between males and females over a 72 hr period ( Zerani et al., 1991). Xenopus are seasonal breeders in the wild with most breeding occurring September (early spring) to March (late summer; Reed, 2005). The extent to which Xenopus follow a seasonal breeding pattern in our lab is not known. However, future studies on amphibians (including *X. laevis*) should evaluate baseline levels of CORT across the year since this varies over the breeding and non-breeding season in both sexes for many mammal species including amphibians e.g., crested newts *Triturus carnifex* ( Zerani & Gobbetti, 1993), aquatic salamanders *Necturus beyeri* ( Nagel et al., 2019) and toads *Rhinella icterica* ( Junior et al., 2024) as dictated by the physiological and behavioural demands of reproduction. Sex differences in CORT may therefore only be evident at certain periods of the breeding cycle.

Reports from both the wild and captivity suggest Xenopus exhibit nocturnal behavioural activity implying CORT levels would be highest during the dark period ( Oishi et al., 2004). However physiological evidence from captive animals is conflicting since Thurmond et al. (1986) reported highest levels of plasma CORT in Xenopus between 0900 and 1200, where-as maximal CORT was excreted during the night from HPI tissue in vitro. Our crude analysis revealed no difference in CORT levels excreted by males or females across the light and dark periods. Repeated sampling across the light and dark period was not conducted in the current study and would better reflect the circadian behaviour of GCs ( Jessop et al., 2014). In our study repeated hourly sampling across the day revealed a non-significant trend of decreasing GCs as observed in many mammalian species across the light period (e.g., Smith et al., 2015). However, based on the data collected to date, we cannot determine if this decreasing trend was of biological relevance and if so if it was due to circadian effects or habituation of the frogs to the transfer and handling process required for sampling. In *R. esculenta*, circadian variations in plasma CORT were only detectable during certain months of the year (i.e. during the breeding season) and the patterning of the rhythms changed across the months ( Dupont et al., 1979). Future validations of waterborne CORT should assess circadian rhythms across the year and assess more subtle changes in CORT across the light and dark periods in order to reliably interpret CORT results and plan time frames for experiments.

We found no relationship between SVL and CORT levels similar to Gabor et al. (2013) (but in contrast to Millikin et al., 2019). Species differences in relationships between morphology and physiology should guide methodological processes in how body and CORT measurements are taken in addition to fundamental biological differences. To our knowledge there is minimal published data reporting a correlation between SVL and CORT levels despite many studies correcting for this body measure e.g. San Marcos salamander *Eurycea nana,* and the common midwife toad *Alytes obstetricans* ( Gabor et al., 2013); red-spotted newts ( Aspbury et al., 2017); various *E subspecies* ( Gabor et al., 2016), and tungra frogs ( Baugh et al., 2018). Some researchers argue the importance of applying body size correction factor (e.g. Scott & Ellis, 2007) although we question the scientific validity of this when there is no proven relationship. Further research should assess alternative methods for controlling for any potential impact of body physicality such as a body condition index score ( MacCracken & Stebbings, 2012; Ruthsatz et al., 2023b) or surface-area-to-volume ratio ( Ferreira Amado et al., 2019), especially since body size will be confounded by sex in sexually dimorphic species such as *X. laevis.*

Amphibians are an imperilled phyla ( IUCN, 2024) due to climate change, habitat destruction, invasive species, over exploitation, pollution and increased exposure to infectious disease such as chytridiomycosis ((Bd) ( Blaustein et al., 2011; Ford et al., 2020; Palomar et al., 2023). Around 41% of amphibian species are threatened with extinction ( Alroy, 2015; Green et al., 2020; Wake & Vredenburg, 2008). Validated tools are urgently required to monitor HPI function in wild amphibians to understand what impacts HPI function in wild populations, predict responses to environmental change, identify compromised groups, inform management decisions and monitor the impact of conservation mitigation strategies ( Breuner et al., 2008; McClelland & Woodley, 2021; Narayan et al., 2019; Ruthsatz et al., 2023b). Measurement of waterborne CORT is already positioned as an important tool in the Conservation Physiology ‘toolbox’ for monitoring GCs in aquatic organisms in both the lab and the field ( Madliger et al., 2018). We hope our methods will be useful for practitioners designing experiments using waterborne CORT in conservation focussed studies in the lab and in the field.

Methods measuring waterborne CORT are still in their infancy and many unknown parameters remain that require clarification ( Ruthsatz et al., 2023b). Below we identify some of the limitations to the methods which we hope can be resolved with further research.

1. The potential welfare impacts of singly housing frogs in a water that is different to the home tank water have been discussed above. Our recent work ( Moss et al., 2024) showing that waterborne CORT can be reliably measured in tank water used in recirculating systems for housing Xenopus are promising and warrant further research.
2. Our method described here is only validated for singly housed adults and not group housed adults which could have welfare implications for a social species such as *X. laevis.* The application of our methods to groups of animals would reduce individual sampling stress, may reduce data variability since a single hormone concentration would be the net result of CORT values from several animals and allow questions to be answered at the level of a group (although not at the level of the individual). However, in small amphibians such as tadpoles, there is the risk of individuals absorbing excreted CORT from tank members during sample collection through their permeable skin membranes confounding individual HPI activity, feedback loops and release rates ( Forsburg et al., 2019; Wack et al., 2010). This also has implications for rearing conditions since small amphibians reared in groups may have altered HPI functioning due to the absorption of exogenous CORT during development compared to singly raised animals ( Crespi, & Warne, 2013; Ruthsatz et al., 2023a) confounding CORT measures. We encourage future research assessing the applicability of our methods to quantifying CORT in groups of aquatic animals taking into account sociality and the size of the subjects ( Forsburg et al., 2019; Narayan et al. 2013).
3. There are several potential sources of CORT from the frog (e.g., faeces, urine, saliva, and passage via the skin and gill membranes) and we do not know the relative contribution of these to the CORT value we are obtaining. Furthermore, the ratios of CORT released from each source may vary depending on intrinsic and extrinsic factors such as stress status and/or developmental stage. For example, if urinary CORT forms a major component of the waterborne CORT measured, varying urine dilutions will confound CORT measures unless samples are controlled for urine dilution e.g. by measuring levels of creatinine in the urine – a typical metabolic by-product used to control for urine dilution in mammals ( Burtis & Ashford, 2001).
4. The clearance rates of CORT to excretion via urine, faeces, saliva and dermal secretions may vary across conspecifics in different developmental conditions. For example CORT excretion via the skin and gills will probably change across development with increasing skin keratinization ( Nishikawa et al., 1989; Tata, 2006) and gill degeneration ( Burggren & West, 1982) confirming that waterborne CORT measurement methods require validation across individual life stages ( Ruthsatz et al., 2023b).
5. Waterborne CORT has been validated as an indicator of pollution stress in amphibians e.g. *R. temporaria tadpoles* ( Ruthsatz et al., 2023a). However, if pollutants damage the skin ( Fenoglio et al., 2009) or gills ( Bernabò et al., 2013) this will impact CORT excretion and confound levels of waterborne CORT measured. We advocate careful selection of and robust validation of stress biomarkers to minimise confounds arising from pollutants. Combining waterborne CORT measurements with behavioural indicators of well-being (especially those known to be impacted by CORT in amphibians such as feeding rates and locomotion, Crespi, & Denver, 2004) or down-stream measures of stress such as body condition and growth rate ( Breuner et al., 2013), may be effective to assess the impact of pollutants on amphibians (for example reduced foraging levels and growth rate were associated with high levels of salinity in the larvae of wood frogs ( *Lithobates sylvaticus*) in the natural habitat ( Hall et al., 2017).
6. The methods described here are tailored to quantifying waterborne CORT rather than *cortisol* yet growing evidence suggest cortisol is also an important GC in amphibians ( Westrick et al., 2023). Current methods for quantifying waterborne cortisol in amphibians use broadly similar collection and steroid extraction methods to us (e.g. Westrick et al., 2023) hence we expect our work to also be of relevance to those measuring cortisol although this needs to be evaluated.
7. GC release in vertebrates is confounded by numerous non-stress related endogenous physiological factors such as sex, age, reproductive status, social rank among many, which confound GC measures ( Millspaugh & Washburn, 2004; Palme, 2019). In amphibians GC levels change with reproductive status, ( Nagel et al., 2019; Junior et al., 2024), age ( Ruthsatz et al., 2023b) and body condition ( Ruthsatz et al., 2023b) but further work is required to examine the full extent to which physiological parameters other than stress impact GC levels in amphibians so these can be controlled for.

## Conclusions

We report a series of robust validations for our non-invasive method of measuring waterborne corticosterone in *X. laevis.* These methods may be adapted for use in different captive and wild aquatic amphibian species to optimise husbandry procedures and contribute to the conservation of amphibians, for whom the existence of many species in the wild is severely threatened ( Green et al., 2020).

### Ethical considerations

All the procedures conducted in consultation with the UK Home Office, in accordance with University of Chester Research Guidelines and under approval from the University of Chester Faculty Research Ethics Committee (FREC ref no 986/14/CH/BS 25 Nov 2014).

## Data Availability

The underlying data have been deposited in ChesterRep. Validating the underpinnings of water corticosterone measurement for aquatic amphibians. http://hdl.handle.net/10034/629000 ( Smith et al., 2024a). This project contains the following underlying data:
• Raw data Smith et al.xlsx (Raw corticosterone data from each experiment in addition to the identifiers for animals used in each experiment and their weights at the start of each experiment). Data are available under the terms of the Creative Commons Attribution 4.0 International license (CC-BY 4.0). The ARRIVE guidelines 2.0: author checklist have been reported in ChesterRep. http://hdl.handle.net/10034/629044 ( Smith et al., 2024b).
• TS2Authorchecklist-full.pdf (The ARRIVE guidelines 2.0: author checklist including ‘The Recommended Set’). Data are available under the terms of the Creative Commons Attribution 4.0 International license (CC-BY 4.0).
