# Supplementary material for: Validating the underpinnings of water corticosterone measurement for aquatic amphibians
Source: F1000Res. 2025 Jan 9;14:53. [Version 1] doi: 10.12688/f1000research.157055.1 (PMC11865716; doi:10.12688/f1000research.157055.1)
Supplement: Peer review history

# Reviewer report: Reviewer response for version 1

**DOI:** 10.5256/f1000research.172458.r362100

**Published:** 2025-02-26

**Authors:** Nikolett Ujhegyi

**Affiliations:** 1 HUNLREN Centre for Agricultural Research, Budapest, Hungary

This manuscript validates a non-invasive method for measuring waterborne corticosterone in Xenopus laevis to assess amphibian welfare, finding that deionized water minimizes background corticosterone, the filtration process does not alter hormone levels, repeated sampling does not increase stress, and corticosterone levels are unaffected by circadian rhythm, sex, or body size, establishing a reliable tool for welfare monitoring in aquatic amphibians.

 This is a very valuable study that presents a summary and comprehensive methodology for measuring non-invasive stress hormone. Of particular note is that it also measured and compared background hormone levels and discussed the important role of hormone sampling fluid types. The methodological results obtained can be interpreted for other species as well as for field conditions. I have only made a few minor comments and suggestions on the manuscript.

 General comments:

- Perhaps it would be may better to give the actual unit of measurement on the y-axis in the figures, not just "sample", because the unit of measurement was different for each sampling type. Or if it was standardized to L at the end then write that down.
- Consistency of terminology: for example, Xenopus laevis in italics. In the first paragraph of the introduction, please write the correct scientific name of the species "Xenopus laevis (Daudin, 1802)" in the first occurrence. If you abbreviate Xenopus to X., please insert () in the first or second occurrence to indicate the abbreviation used later
- Page 21 5 a parenthesis is missing from the end of the sentence. "Combining waterborne CORT measurements with behavioral indicators of well-being (especially those known to be impacted by CORT in amphibians such as feeding rates and locomotion, Crespi, & Denver, 2004)").

Some methodological comments and questions:

- Page 5 you write this: “(10) several authors have shown levels of waterborne CORT to be highly repeatable across time”, but you cite only one article whom researched 2 species. Proposes to transcribe "some authors", or you should cite reviews.
- Page 8: You write this "When possible, repeated measures samples from a single frog and samples from a single experiment were analysed on the same enzyme immunoassay plate to reduce within frog and within experiment variation". I am not sure that this would always be the best method. If we eliminate the plate effect, we can systematically bias our samples in some direction. Did you somehow correct for this possible bias in the analysis, or did you account for it somehow?
- Page 9: Interestingly, background CORT was also measured in 5 different control water type. What could be the reason for measuring CORT in type 5 when it was filtered, "cleaned'- standard" dechlorinated water that was filtered twice and passed through a primed Sep-Pak column?
- Page 10: 800 pg/L seems a bit high (at least to me), is there any literature on what the CORT level is in tap water and other waters there? If there is, it might be worth putting in a citation for that as well.
- Page 10 Ai3: I don't quite understand why a control group hasn't been done here? If e.g. the filtering technique consistently takes away from the measured CORT, it would have been more worthwhile to have a control water sample spiked with CORT, because then the real possible CORT loss can be better measured. Could you explain a little more why you did it that way? In addition, in the case of Ai33, there were only 4 females in the holding water instead of 8. It would be nice more detailed explanation of this even in the manuscript. 8 animals vs 4 animals may not excrete the same amount of CORT, so it is also harder to compare. It would be lovely if there was a flowchart in the manuscript for better clarity and visibility, if possible.
- Page 11: A3 result. This is an interesting result; the data should be included in a supplement, and a figure illustrating this result would be very useful in the manuscript. Could you explain it more why used you eight females in Ai but only four in Aii?
- A4 method. It's unclear why exactly 48 hours were chosen; if the amount of water increased fivefold, why did the elapsed time change so dramatically? This should be explained more clearly in the manuscript.
- The A4 result is also interesting. How could CORT increase over time if there were no animals involved? Could it be that CORT was not homogeneous across the large sample, resulting in different amounts in the subsamples? Or could they only measure these on different plates, and the results could be influenced by sampling bias? Every scatterplot contains a significant outlier value; what were these? Did they originate from the same tank?
- Maybe it just escaped my attention, but in B3 methods: could you please elaborate on when and how the animals were fed during the 48-hour experiment? Ad libitum? Were they fed before the experiment or during the 48-hour period? It would also be worthwhile to measure the body size of the control individuals, who are not part of the experiment but are fed in the same time as the experimental animals.

Thank you for the valuable manuscript, which was very well written.

Is the rationale for developing the new method (or application) clearly explained?

Yes

Is the description of the method technically sound?

Yes

Are the conclusions about the method and its performance adequately supported by the findings presented in the article?

Yes

If any results are presented, are all the source data underlying the results available to ensure full reproducibility?

Partly

Are sufficient details provided to allow replication of the method development and its use by others?

Yes

Reviewer Expertise:

evolutionary biologists, behavioral ecologists

I confirm that I have read this submission and believe that I have an appropriate level of expertise to confirm that it is of an acceptable scientific standard.

# Reviewer report: Reviewer response for version 1

**DOI:** 10.5256/f1000research.172458.r359834

**Published:** 2025-02-10

**Authors:** Sarah E. Westrick

**Affiliations:** 1 Biology, Wake Forest University Department of Biology (Ringgold ID: 124555), Winston-Salem, North Carolina, USA

This study is a comprehensive set of experiments to validate methodological variables relevant to measuring corticosterone in water samples from aquatic amphibians. The study addresses (1) the type of water used, (2) the impact of using plastic vs. glass collection containers, (3) the impact on filtration of samples on CORT levels, (4) how CORT degrades in water samples kept at room temperature over time, (5) the impact of repeated handling and transfer of frogs on CORT levels, (6) variation in CORT between light and dark cycles, (7) sex differences in waterborne CORT, and (8) the relationship between body size (snout-vent length) and waterborne CORT. 

 This is a valuable study for the field! I appreciate the thoroughness and the amount of work the authors put into making this an extensive assessment of methodological concerns in collecting waterborne hormones. The paper is well-written and clear and will be a useful reference for future studies. Any questions or hesitations about the data were appropriately interpreted and cleared up in the thorough discussion section. I only have two minor edit suggestions.

 1) In the introduction, the authors suggest fish are "higher" on the taxonomic ladder. I believe bony-fish fish (as a group) are typically considered more 'basal' than amphibians. 

 2) In the introduction, Westrick et al. 2023 suggest cortisol is more abundant in poison frogs but not necessarily more dominant (depending on how dominance is defined).

Is the rationale for developing the new method (or application) clearly explained?

Yes

Is the description of the method technically sound?

Yes

Are the conclusions about the method and its performance adequately supported by the findings presented in the article?

Yes

If any results are presented, are all the source data underlying the results available to ensure full reproducibility?

Yes

Are sufficient details provided to allow replication of the method development and its use by others?

Yes

Reviewer Expertise:

behavioral neuroendocrinology with a focus on glucocorticoids

I confirm that I have read this submission and believe that I have an appropriate level of expertise to confirm that it is of an acceptable scientific standard.
